# Relationship between gut environment, feces-to-food ratio, and androgen deficiency-induced metabolic disorders

**DOI:** 10.1080/19490976.2020.1817719

**Published:** 2020-09-29

**Authors:** Naoki Harada, Yukari Minami, Kazuki Hanada, Ryo Hanaoka, Yasuyuki Kobayashi, Takeshi Izawa, Takashi Sato, Shigeaki Kato, Hiroshi Inui, Ryoichi Yamaji

**Affiliations:** a Division of Applied Life Sciences, Graduate School of Life and Environmental Sciences, Osaka Prefecture University, Osaka, Japan; b Division of Veterinary Science, Graduate School of Life and Environmental Sciences, Osaka Prefecture University, Osaka, Japan; c Institute for Molecular and Cellular Regulation, Gunma University, Maebashi, Japan; d Graduate School of Science and Engineering, Iryo Sosei University, Fukushima, Japan; e Division of Clinical Nutrition, Graduate School of Comprehensive Rehabilitation, Osaka Prefecture University, Osaka, Japan

**Keywords:** Apparent digestibility coefficients, feces, *Firmicutes*/*Bacteroidetes*, gut microflora, longevity, metabolic syndrome, sarcopenic obesity, stool, testosterone, type 2 diabetes mellitus (T2DM)

## Abstract

Androgen action generates sex-related differences that include changes in the gut microbiota composition. Hypoandrogenism and hyperandrogenism in males and females, respectively, are associated with the prevalence of metabolic disorders. Our recent work showed that male androgen receptor knockout (ARKO) mice developed high-fat diet (HFD)-dependent sarcopenic abdominal obesity, hyperglycemia, and hepatic steatosis, leading to early death. The ARKO mice also exhibited alterations in intestinal microbiota but did not experience metabolic abnormalities when administered with antibiotics. Here, we show that time-dependent changes in feed efficiency (ratio of body weight gain to food intake) and weight of dried feces-to-food ratio could be good markers for changes in gut microbiota. *Turicibacter* spp., *Lactobacillus* spp., and *L. reuteri* increased in the gut in both HFD-fed ARKO and castrated mice having metabolic abnormalities. HFD-fed ARKO mice showed increased plasma levels of aspartate, but not alanine, aminotransferase. Changes in the gut microbiome appear to provoke androgen deficiency-induced metabolic diseases, leading to early mortality.

Androgens underlie many sex-related differences in the development and function of both reproductive and non-reproductive tissues. Testosterone levels temporarily rise during male fetal development,^1^ and this produces sex-related differences at birth. Fetal androgen not only acts to promote the development of reproductive organs such as internal and external genitalia but also acts on the development of non-reproductive organs such as pancreatic β-cells in males.^2,3^ A transient testosterone surge in neonates is responsible for brain masculinization.^1^ Additionally, an increase in testosterone during puberty contributes to the development of secondary sexual characteristics in males.^1^ The action of androgen has different effects on metabolic diseases in men and women. Hypoandrogenism and hyperandrogenism in males and females, respectively, are associated with metabolic disorders.^4^ In this paper, we will discuss the involvement of the gut microbiome in androgen-related metabolic disorders.

## Role of androgens in sexual maturation and sex-related differences in gut microbiota

In humans, the gut microbiome is composed of ~4 × 10^13^ bacteria (~0.2 kg) with ~1000 different species described.^5,6^ Sex-related differences have been reported in the composition of gut microbiota for both rodents and humans.^7–16^ Decrease in α-diversity of microbiota in the gut is associated with obesity,^17,18^ and α-diversity is lower on average in males compared to females,^10–12,14^ especially in young adults (ages 20 to 45 years) compared to middle-aged adults.^10^ The effect of sex on gut microbiota composition is larger than that observed in response to a high-fat diet (HFD) or treatment with antibiotics,^8^ but is smaller than effects due to genotype.^13^

While the composition of gut microbiota is indistinguishable between both sexes during the pre-pubertal period, differences become apparent after sexual maturity has been reached.^14,15^ Compared to sexually mature males, the gut microbiome composition of sexually mature females is more similar to that of sexually immature mice, and that of castrated males is more similar to that of females.^14^ This demonstrates the important role of pubertal androgens in the formation of sex-dependent differences that are observed in gut microbe compositions. These sex-dependent differences in the gut microbiome also have an influence on the prevalence of diseases that show a sex bias. It has been clearly shown that type 1 diabetes exhibits a female bias in NOD mice,^14,15^ and that the gut microbiome in mature male NOD mice is protective against the disease.^14,15^ Sex differences in gut microbiota are also suggested to play a role in the development of other diseases that show a sex bias, including rheumatoid arthritis,^19^ anxiety disorder,^20^ hepatocellular carcinoma,^21^ and inflammatory bowel disease.^11,22^ Sex-by-diet interactions in gut microbiota have been observed in C57BL/6 J mice^7,8^ and humans,^9^ and gut microbiota play a critical role in HFD-induced obesity and insulin resistance.^23^ The combinatorial effects of androgen and diet may therefore induce pathological conditions that have a sex bias through modulation of gut microbiota.

## Androgens and gut microbiota composition in metabolic disorders in males

Low androgen levels are a risk factor for metabolic syndrome, type 2 diabetes, cardiovascular disease, and early death.^1,24^ Androgen deprivation therapy for prostate cancer patients also causes metabolic disorders^1^ and affects gut microbiota composition,^25^ suggesting that gut microbiota are involved in androgen deficiency-induced metabolic abnormalities. To test this hypothesis, we utilized castration and androgen receptor (AR) knockout (ARKO) mouse models. AR, a member of a nuclear receptor superfamily of ligand-dependent transcription factors, mediates the action of androgen. Thus, the physiological functions of androgen can be experimentally determined in laboratory animals through castration and ARKO models. We first demonstrated that castration causes metabolic disorders that are involved in cardiovascular events including abdominal obesity, hyperglycemia, hepatic steatosis, and thigh muscle loss when on an HFD.^24,26^ Our subsequent recent study with ARKO mice also revealed that all these metabolic disorders were observed in male mice fed an HFD.^27^ Notably, these metabolic disorders were abolished when mice were administered a cocktail of antibiotics in both castration and ARKO models.^26,27^ Below, we report additional data from the ARKO mouse study and identify similarities and differences between the castration and ARKO models.

**Figure 1.**
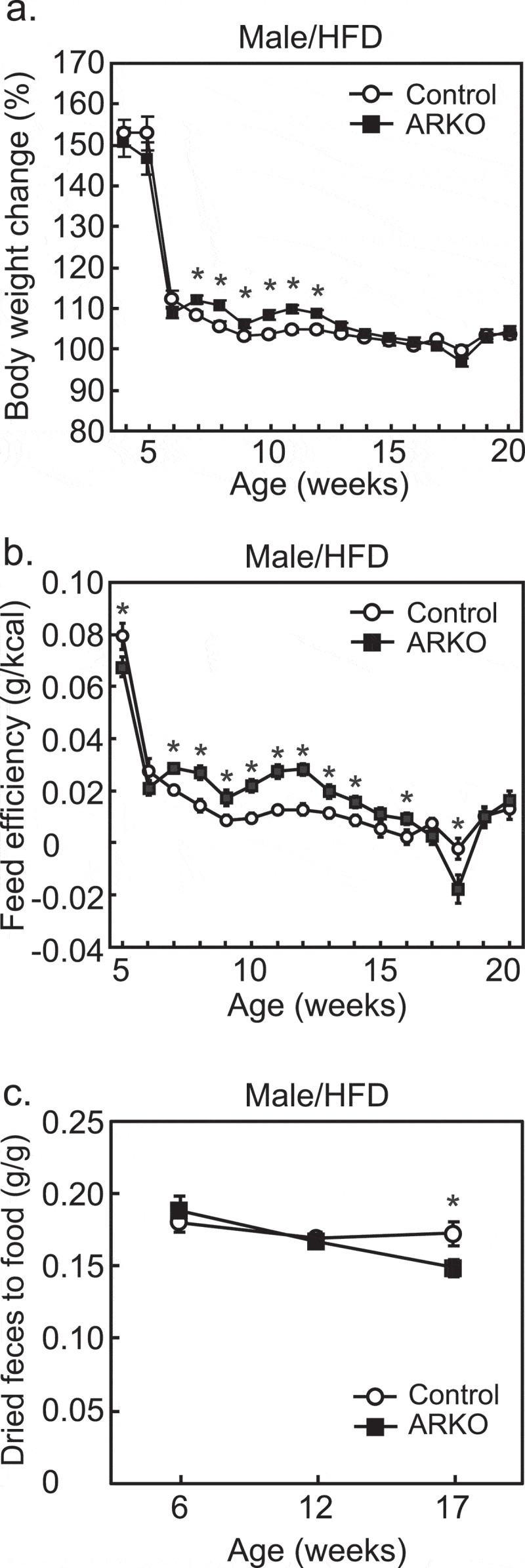
Effects of androgen receptor knockout (ARKO) on body weight gain, feed efficiency, and digestive efficiency in male mice fed a high-fat diet (HFD). Mice were fed an HFD up to 20-weeks of age, as previously described.^27^ (a) Changes in body weight (%) and (b) feed efficiency (body weight gain/food intake) were determined once a week (control, n = 16; ARKO, n = 19). (c) Digestive efficiency (dried fecal weight/food intake) was determined at 6, 12, and 17 weeks (n = 11). Data were expressed as mean ± SEM, and the threshold for statistical significance was set at *p* < .05, *.

As previously reported,^28,29^ we observed an HFD-dependent late-onset obesity in male, but not female, ARKO mice.^27^ The percentage of body weight gain with an HFD, over 7 to 12 weeks of age, was higher for the male ARKO group than the control group (Figure 1(a)). This was accompanied by increased feed efficiency (body weight gain/energy intake) without hyperphagia from 7 to 16 weeks of age (Figure 1(b)). The feed efficiency was not increased after 17 weeks. The reason for this is not clear. However, experimental procedures involved in glucose and insulin tolerance tests, which were started at 16 weeks, could affect the efficiency. Of note, similar results were also observed in the castration model.^26^ In contrast, we observed a decrease in the ratio of fecal weight to food intake in the ARKO group at 17, but not at 6 and 12 weeks of age (Figure 1(c)). The kinetics of these changes suggested that the decrease in fecal weight is not the cause of increased feed efficiency, but the result of metabolic changes. Similar to the ARKO model, castrated obese mice on an HFD also showed a decrease in the ratio of fecal weight to food intake.^26^

Alterations in gut microbiota composition affect up to 2% of total energy intake from food consumed.^30^ If the average daily energy intake is 2,000 kcal, 2% would correspond to 40 kcal, and a consistent surplus would account for up to 14,600 kcal (40 kcal × 365 days) per year. This value corresponds to a gain of ~2 kg of adipose tissue, of which 80% is comprised of fat (9,000 kcal/kg). This estimate emphasizes the importance of gut microbiota in energy homeostasis. The changes in food energy intake strongly suggest that fecal waste is affected by gut microbiota composition. Apparent digestibility coefficient (ADC) is used to measure the digestion of nutrients. ADC accounts for several factors, such as dry matter, organic matter, crude protein, amino acid, lipid, carbohydrate, or digestible energy, and can be measured and calculated using the following formula: ADC (%) = (ingested – fecal)/(ingested) × 100. We propose that where the same diet is used, this concept (*i.e*., the ratio of fecal matter to ingested matter) can be used to estimate changes in gut microbiota. As feces is composed of 70–80% water,^31^ measurement of weight of food intake and dried feces is a simple method (Figure 1(c)) that can be used to indicate changes in the gut environment, including gut microbiota composition.

**Figure 2.**
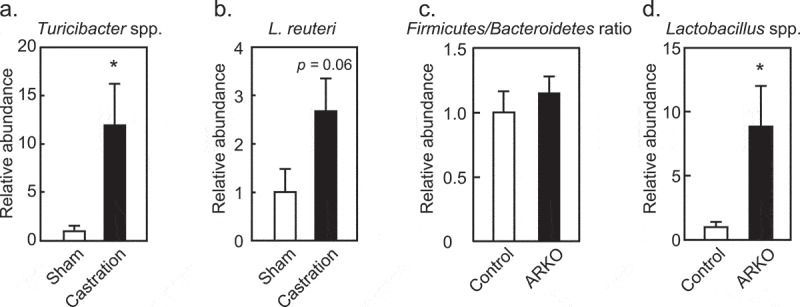
Effects of androgen receptor knockout (ARKO) or castration on fecal microbiota of male mice fed a high-fat diet (HFD). In castration and ARKO models, mice were fed an HFD, as previously described.^26,27^ Feces were collected at 13 (castration) or 17 (ARKO) weeks of age and extracted DNA was analyzed by real-time PCR with specific primers.^26,27^ Relative abundance of (a) *Turicibacter* and (b) *Lactobacillus reuteri* in the castration model (HFD sham, n = 8; HFD castration, n = 9). (c) *Firmicutes* to *Bacteroidetes* ratio, and (d) *Lactobacillus* species in the ARKO model (n = 11) is also represented. Data were expressed as mean ± SEM, and the threshold for statistical significance was set at *p* < .05, *.

Gut microbiota was involved in metabolic dysregulation observed in castrated (post-pubertal)^24,26^ and ARKO^27^ models. Thus, we analyzed gut microbiota changes in these two androgen deficient models for commonalities based on their total abundance and the rate of change previously observed in a metagenomics analysis of ARKO^27^ and castration^24,26^ models. Increase in *Turicibacter* and *Lactobacillus reuteri* that were characteristic of ARKO mice^27^ were also observed in castrated mice on an HFD (Figure 2(a,b)). In contrast, increase in the *Firmicutes*/*Bacteroidetes* ratio in castrated mice on an HFD^26^ was not observed in ARKO mice on an HFD (Figure 2(c)). The increase in genus *Lactobacillus* in castrated mice^26^ was also seen in ARKO mice (Figure 2(d)). As sex differences in gut microbe composition develop during puberty,^14,15^ identifying bacteria common to castrated and ARKO mice is valuable. Increase in *Lactobacillus* and *Turicibacter* was observed for both castrated and ARKO mice on an HFD, and these bacteria are located in the distal small intestine. As such, metabolic abnormalities in castrated and ARKO mice may be developed in the environment of the distal small intestine.

Abundance of phylum *Bacteroidetes* is characteristic of male gut microbe composition in rodents and humans.^11,14,16,32^ An elevated *Firmicutes*/*Bacteroidetes* ratio, which is characteristic of obesity,^33^ was increased in castrated mice, but not in ARKO mice that were fed an HFD. This difference may be due to a requirement of prenatal, neonatal and/or pubertal androgen activity before the onset of androgen deficiency. While high prevalence of metabolic disorders has been observed in males,^34^ androgen deficiencies due to castration or in ARKOs also resulted in metabolic abnormalities in males. These discrepancies suggest that epigenomic differences between males and females during development affect gut microbiota composition.

**Figure 3.**
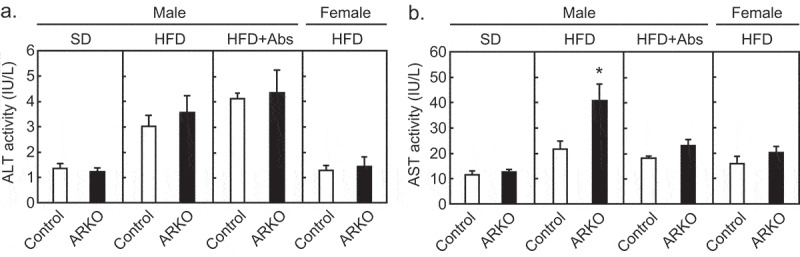
Effects of androgen receptor knockout (ARKO) on plasma transaminase levels in male mice. Control mice and ARKO mice were fed either a standard (SD) or a high-fat diet (HFD) and up to 20-weeks of age, as previously described.^27^ Levels of (a) plasma alanine aminotransferase (ALT) and (b) plasma aspartate aminotransferase (AST) were measured using a Transaminase CII-test Wako (Wako, Osaka, Japan). SD male experimental groups included control (n = 5) and ARKO (n = 6). HFD male experimental groups included control (n = 18) and control treated with antibiotics (n = 9, Abs), ARKO (n = 12), and ARKO treated with antibiotics (n = 10). HFD female experimental groups were control (n = 5) and ARKO (n = 7). Data were expressed as mean ± SEM, and the threshold for statistical significance was set at *p* < .05, *.

Hypogonadism is a risk factor for reduced lifespan in men.^1,35,36^ We observed that the ARKO mice on an HFD had a shorter lifespan compared to the control mice.^27^ Regardless of a fatty liver,^27^ plasma levels of alanine aminotransferase, a specific marker for hepatocellular injury, did not change in the ARKO group fed an HFD (Figure 3(a)). On the other hand, plasma levels of aspartate aminotransferase (AST), a marker of liver, heart, kidney, or muscle injury were increased in ARKO on an HFD (Figure 3(b)). The heart is the potential site of the AST increase, because androgen deficiency is associated with cardiovascular incidents.^1,37^ At the time of death in a lifetime study, the relative heart weight (% of body weight) of the HFD-fed ARKO mice was higher than that of HFD-fed control mice (0.82 ± 0.06 *versus* 1.13 ± 0.05 for the control and ARKO mice, respectively; *p* < .001), whereas the absolute body weight (g) was reduced in the ARKO mice (29.2 ± 1.2 *versus* 22.9 ± 1.3 for the control and ARKO mice, respectively; *p* < .001), and the absolute heart weight (g) was not different between the two groups (0.23 ± 0.015 *versus* 0.25 ± 0.013 for the control and ARKO mice, respectively; *p* = .33). In contrast, at 20 weeks of age, both the relative heart weight (% of body weight) and absolute heart weight (g) of ARKO mice were significantly lower than those of the control mice, irrespective of the diet. Besides, the difference on the age of dead is a limitation, as these results suggest that the heart weight of ARKO mice increased steadily after 20 weeks of age. On the other hand, histopathological examination of the HE-stained paraffin-embedded longitudinally sliced heart tissue revealed focal to multifocal myocardial atrophy, interstitial amyloid deposition, fibrosis, and mononuclear cell infiltration in the hearts of both control and ARKO mice. These findings are known to be sporadically seen in aged mice.^38^ No clear difference was detected in the frequency and degree of these heart lesions between the control and ARKO mice at the time of death. Therefore, future studies are needed to determine which organ is responsible for the increase in AST and understand the involvement of cardiac failure in the premature death of ARKO mice observed in our current study.

## Effect of gut microbiota on blood androgen levels

Testosterone levels differ in germ-free and specific pathogen-free mice,^15^ suggesting that gut microbiota regulate blood testosterone levels. Other studies have also provided several lines of supporting evidence for this. Testosterone levels are modulated in gnotobiotic mice (mice that have defined microbiota),^14,15^ and colonization by certain bacteria, including segmented filamentous bacteria, elevates blood concentrations of testosterone.^14^ Specific bacteria, such as *Clostridium scindens*, can synthesize androgens from glucocorticoids.^39^ Other strains of bacteria have 5α-reductase activity,^40^ which enables them to produce the most potent androgen, dihydrotestosterone, from testosterone. Some gut microbiota regulate androgen levels in the intestine by deglucuronidation to release free dihydrotestosterone from its glucuronide conjugates.^41^ This also demonstrates the ability of gut microbiota to regulate androgen levels through different mechanisms.^41^ Conversely, bacterial–host interactions can also be regulated by androgen levels.^26,42,43^

## Effect of androgens on gut microbiota in females

Hyperandrogenism is a criterion for polycystic ovary syndrome (PCOS), a heterogeneous disorder that has a global prevalence of 5–15% in females and involves metabolic dysregulation.^32^ Changes in gut microbiota and reduced α-diversity in PCOS patients have been reported.^32,44^ Notably, the transplantation of fecal microbiota has been reported to be beneficial for PCOS.^45,46^ These results indicate that androgen-related gut microbiota changes are involved in PCOS pathophysiology. Fetal or neonatal androgen excess is also associated with dysbiosis in a model of PCOS,^47,48^ supporting the idea that fetal and neonatal development affects long-term gut microbiota composition.

## Conclusion

**Figure 4.**
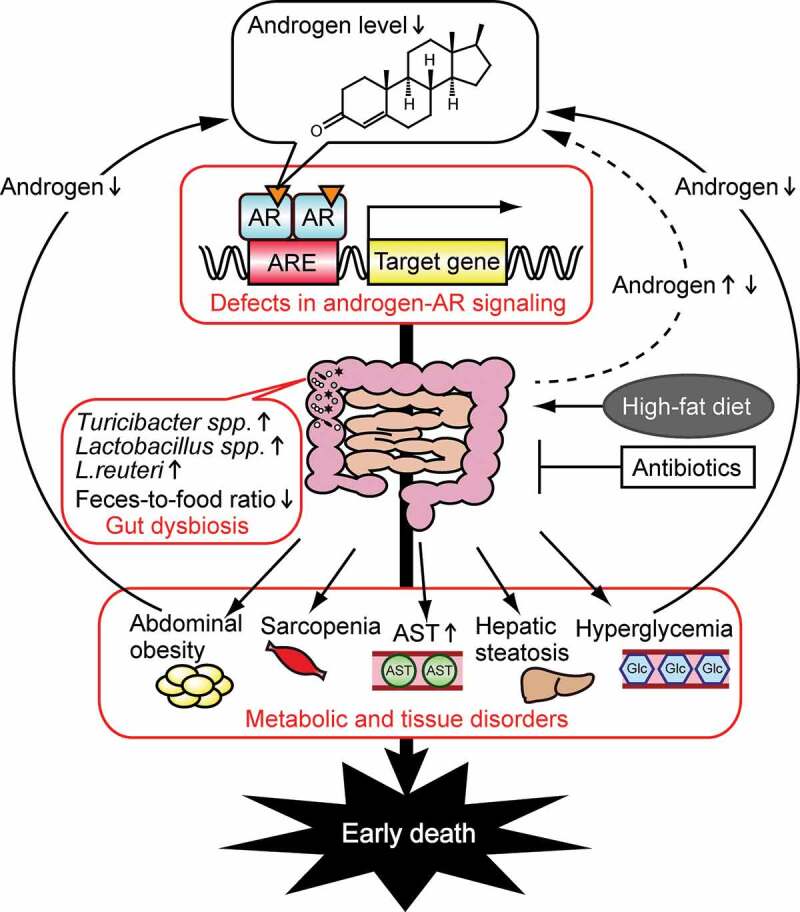
Schematic of the effects of androgen on metabolic disorders in males. Defects in androgen–androgen receptor (AR) signaling (including castration and ARKO) cause gut dysbiosis, result in abdominal obesity, hyperglycemia, hepatic steatosis, sarcopenia, and also increase aspartate aminotransferase (AST) levels, leading to early death.^24,26,27^ Androgens levels are decreased by obesity and hyperglycemia, and are regulated by gut microbiota. ARE, androgen response element; Glc, glucose.

The sex differences observed in metabolic homeostasis^34^ can at least in part be explained by differences in the gut microbiome. Androgens are responsible for the sex differences in gut microbes, which we found to play pivotal roles in pathologies associated with hormone insufficiency or excess in both males and females. As summarized in Figure 4, androgen deficiency-induced dysbiosis causes metabolic disorders in male mice when fed an HFD, and is associated with shorter lifespan. Among metabolic disorders, obesity and hyperglycemia negatively impact testosterone levels.^1,49^ Defects in androgen signaling and related metabolic disorders, therefore, negatively reinforce each other. Hepatocyte ARKO^50^ and neuronal ARKO^51^ mice develop an obese phenotype. However, the role that gut microbiota plays in the obesity of these ARKO mice remains unclear. To elucidate the mechanisms that underlie androgen regulation of gut microbiota composition, future studies are needed to identify target organ(s) that may be involved in androgen-induced alterations of gut microbiota.
